# ‘I Think From the Beginning, the Ambitions Were Compromised’: A Case Study of COVAX as Vaccine Equity Policy Operationalisation

**DOI:** 10.1002/hpm.70028

**Published:** 2025-09-30

**Authors:** Charnele Nunes, Martin Mckee, Simon Rushton, Natasha Howard

**Affiliations:** ^1^ London School of Hygiene and Tropical Medicine (LSHTM) Faculty of Public Health and Policy London UK; ^2^ School of Sociological Studies Politics and International Relations University of Sheffield Sheffield UK; ^3^ Saw Swee Hock School of Public Health National University of Singapore and National University Health System Singapore

**Keywords:** COVAX, global health partnerships, global health policy, innovation, pandemic, vaccine equity, vaccines

## Abstract

**Background:**

COVAX was designed to support the discovery, development, and distribution of COVID‐19 vaccines globally, at scale and pace. This article examines how COVAX promoted vaccine equity and what lessons can be learnt.

**Methods:**

Informed by a scoping review of lessons learnt from GHPs, we reviewed 109 documents related to COVAX and other GHPs and conducted 23 key informant interviews with representatives from GHPs, civil society, academia, and the private sector. Data were synthesised thematically using Rushton and Williams's framework.

**Results:**

Data showed how the global health policy context shaped COVAX, with experience with Gavi and CEPI influencing its governance structure. We highlighted weaknesses in transparency and accountability, limited engagement with civil society organisations [CSO] and LMIC stakeholders, contested policy debates (e.g., different framing) and paradigms (e.g., prioritising technical and financial over political solutions).

**Conclusions:**

COVAX largely replicated existing GHP approaches, subsidising research and development and then paying for resulting discoveries. While recognising how this reflects global power structures, in the inevitable next global health crisis, the international health community must advocate for greater LMIC and CSO involvement in decision‐making, sharing of intellectual property and technology transfer, and rebalancing of flows of innovation costs and benefits to a broader range of actors across public and private sectors.

## Highlights

- COVAX mirrored existing GHP approaches, diluting its equity goals.
- Global health governance, as currently structured, perpetuates inequalities.
- More inclusive vaccine R&D and decision‐making approaches are needed.
- Policy approaches should balance technical and political solutions.

## 1 Introduction

The onset of the COVID‐19 pandemic highlighted tensions between the interests of the global community collectively and those of individual states. While most governments of rich countries recognised the need for a global response to the pandemic, they struggled to find a means to balance the immediate and obvious needs of their own populations with the distant, and therefore less visible, needs of those in poor countries. The problem became acute in early 2021 when new vaccines, able to transform the pandemic response, became available, but initially in very limited quantities. It was widely agreed that the existing global governance mechanisms were inadequate for the scale of the task [1].

In response, the COVID‐19 Vaccines Global Access Facility (COVAX) was created in 2020 as one of the pillars of the Access to COVID‐16 Tools (ACT) Accelerator. COVAX was a global initiative operating until 2023 to provide equitable access to COVID‐19 vaccines, testing, and therapies [1]. Managed by the Coalition for Epidemic Preparedness Innovations (CEPI), Gavi the Vaccine Alliance, and the World Health Organization (WHO), with UNICEF as delivery partner, COVAX aimed to distribute vaccines fairly across all countries, regardless of wealth [1]. Its funding strategy combined direct investments and volume guarantees to facilitate vaccine production and availability [2]. COVAX's challenges included insufficient vaccine doses, delayed funding and inability to spend when funds did arrive [3], and competing political interests [4], all impeding distribution [5]. Consequently, it delivered approximately 1.2 billion of its two billion‐dose target [6] and while it did disproportionately benefit the poorest countries, this was insufficient to redress the scale of global inequity [7]. Critics argued that COVAX's approach, modelled on Gavi's Pneumococcal Conjugate Vaccine Advance Market Commitment initiative (PCV‐AMC), made only limited progress on vaccine equity, reflecting a lack of incentives for sustainable vaccine innovation and a failure to overcome systemic barriers in global health governance [8].

COVAX adopted a similar funding model to Gavi's pneumococcal vaccine AMC (PCV AMC), but in contrast, employed push funding by providing financing as direct catalytic investment in production facilities as well as pull funding by providing volume guarantees for specific COVID‐19 vaccine candidates before licensure [2, 9, 10, 11]. However, this model did not procure sufficient COVID‐19 vaccine doses and encountered funding shortfalls, vaccine nationalism, and other political barriers [12]. For example, in addition to ongoing support for intellectual property (IP) regimes that limited manufacture, many high‐income countries (HICs) acted to restrict equitable distribution of existing supplies, signing Advance Purchase Agreements (APAs) with vaccine manufacturers to ensure their populations were covered [13, 14, 15]. Though the Advance Market Commitment (AMC) model was reappropriated and redesigned in the COVAX Facility, to reflect specificities of the COVID‐19 pandemic, its design was flawed by not incentivising and supporting long‐term and sustainable vaccine innovations [8, 16]. The model crystallised existing limitations within the vaccine innovation system relating to IP, heavy reliance on the private sector, and limited vaccine manufacturing capacity in LMICs, which served as barriers to achieving global COVID‐19 vaccine equity [5, 17, 18, 19, 20, 21, 22].

In 2021, an Independent Panel for Pandemic Preparedness and Response, convened by the World Health Organization (WHO), called for a new framework to strengthen pandemic preparedness and response [23]. In May 2025, a Pandemic Agreement was formally adopted by the World Health Assembly. It offers a framework for international collaboration and coordination during the pandemic, drawing from lessons learnt from COVAX, particularly in terms of international cooperation to ensure equitable access to pandemic response tools, such as a mechanism for pathogen access, sharing of knowledge and benefit sharing [24]. However, many elements have yet to be agreed upon, so as negotiations continue, it will be important that they are informed, to the fullest extent possible, by the lessons that can be learnt from the pandemic [24].

We thus aimed to explore how COVAX promoted vaccine equity and lessons that could be applied to future vaccine development and distribution initiatives during global health crises. The study's objectives were to: (i) describe how the global policy environment influenced COVAX development and operationalisation; (ii) explore how well COVAX promoted global vaccine equity; and (iii) identify lessons for future vaccine equity initiatives.

## 2 Methods

### 2.1 Study Design

We conducted an exploratory single case study using a constructivist international relations (IR) lens and data from document analysis and semi‐structured interviews with academics, policymakers and logistics experts involved in COVAX planning and delivery, informed by findings from a scoping literature review [25]. Our paper has been prepared in conformity with the Consolidated Criteria for Reporting Qualitative Research (COREQ) checklist and Guidance for Reporting Involvement of Patients and the Public Short Form (GRIPP2‐SF), as well as the standards for secondary qualitative analysis. Our choice of theoretical lens recognises the role of multiple actors in supranational policymaking, including global health partnerships (GHPs) in the global vaccine ecosystem. Using Rushton and Williams' maximalist definition of global health policy as ‘those policies, both formal and informal, adopted on either an international or domestic level that respond to or affect health’ [26], we argue that COVAX embodied policies to promote global vaccine equity and explore the structural factors that influenced its ability to achieve its goal.

**FIGURE 1:**
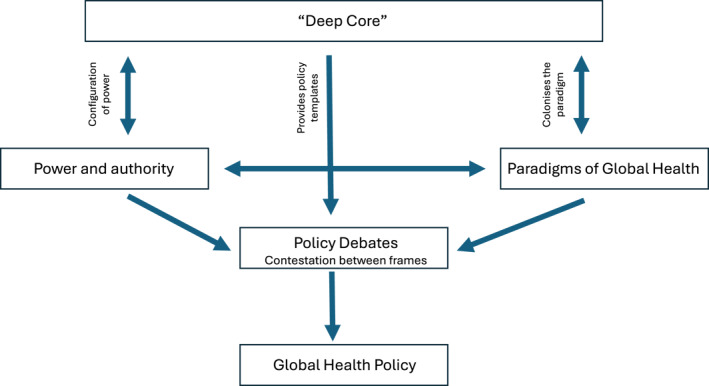
Rushton and William's global health policymaking framework. *Source:* Rushton and Williams [26].

Figure 1 illustrates the constructivist framework we used, based on our literature review, to conceptualise the processes through which COVAX, as a component of global vaccine policy, was developed, as well as the mix of power, framing, paradigms, and neoliberal ‘deep core’ that influenced its design and, ultimately, its effectiveness. Much of the existing literature has identified a range of factors that explain the ‘failure’ of global health governance, but it does not sufficiently engage with the global health policy processes, creating a risk that this failure will be perpetuated [26]. We used this framework to understand core determinants influencing COVAX's global vaccine equity achievements and whether this goal was ever possible in a neoliberal environment (an issue arising in our scoping review [25]) and to structure our analysis and recommendations.

Our research question was: ‘How did different actors and ideas affect the design and operationalisation of COVAX?’

### 2.2 Data Collection

#### 2.2.1 Document Review

The objective of the document review was to collect material from relevant sources to generate a clear understanding of the policies and policy environment related to the COVAX Facility. CN purposively searched publicly available documents from the repositories of COVAX's partners (i.e., CEPI, Gavi, WHO, UNICEF) that were published between April 2020 and December 2021, related to how COVAX described itself (i.e., COVAX's organisational development, governance, and policy operationalisation, activities, and outcomes). The choice of dates for inclusion aligned with the launch of COVAX in 2020. All types of documents were included. We extracted and synthesised data from 109 eligible policy documents, technical/informational reports, terms of reference, board and committee meeting minutes, and distribution forecasts using the READ framework developed by Daglish, Khalid and McMahon [27].

#### 2.2.2 Interviews

We developed an interview guide based on our previously published review of global health partnerships [25] and our initial analysis of the documentary material. Together, these provided insights into agenda‐setting and decision‐making processes at the global level, policy creation within COVAX, and vaccine equity during the COVID‐19 pandemic. The interviews were used to confirm or refute findings from the document review and seek explanations for why issues were addressed or not. The literature review was updated to inform the writing of this paper in May 2023.

We purposively sampled policymakers, academics, and civil society organisation (CSO) representatives based on their current or past involvement with ACT‐A, COVAX, or vaccine GHPs. Academics included researchers from universities and think tanks; policymakers included individuals engaged in national or supranational policymaking; and CSOs included representatives of NGOs, activist groups, and professional associations.

We invited 37 participants for an interview via email from a seed list developed through online searches. Twenty‐eight initially agreed to participate, but due to administrative and scheduling problems, only 23 were eventually interviewed. All gave written informed consent. CN conducted all 23 interviews via Zoom, between February and April 2022, providing a choice of English or French (all chose English). Each interview lasted approximately 35 min (range 30–45) and was transcribed verbatim by CN, with no repeat interviews conducted. During the interviews, only CN and the participant were present in the video call. The interviewer (CN) was a doctoral student at the time of the interviews, experienced in qualitative methods, and had no prior personal relationships with the participants. Although we were unable to interview all those invited, we achieved a broad range of participants and data saturation, as assessed by the richness of the data gathered, similarity of responses, and absence of new ideas emerging, so we did not seek to recruit replacements [28].

### 2.3 Analysis

CN used thematic analysis [29], analysing document data abductively to identify core meanings, and interview data inductively to crystallise findings or identify cleavages between what participants said and what was reported in literature and documentation. Thematic analysis of the documents was conducted manually. Initial coding, based on our objectives and framework, was continued and expanded iteratively during the interviews, exploring comparisons and evidence of convergence, complementarity, and divergence. New codes were added when no existing codes fit the data point in question. Codes were clustered into themes to detect patterns of frequency and interrelationships and organised according to framework categories. Given the ongoing interplay between data collection and analysis, no analytical distinction was made between data from different sources, so findings are presented together.

Interviews were transcribed using Microsoft Word for Mac (version 16.43). Following each interview, transcription and coding were conducted to generate and refine themes. The coding scheme primarily emerged through inductive approaches, informed by the interview guide, literature review, and analysis of the interviews. Interview transcripts were also analysed inductively using a reflexive thematic analysis. approach.

A complete list of codes was developed during the interviews and the entire analysis process following data collection. CN discussed and reviewed the code list with MK and NH, and new codes were added when none of the existing codes fit the data in question. The descriptive codes were then clustered into categories to detect patterns of frequency and interrelationships. During the data analysis process, a taxonomy of themes was developed based on the clustering of codes described above. The overarching ‘parent’ themes were established based on the coding in line with the research questions and theoretical framework, particularly the keywords used in that process.

### 2.4 Ethics

The LSHTM Observational Research Ethics Committee provided ethics approval (reference 26297).

## 3 Findings

Table 1 summarises the types of documents reviewed, highlighting the predominance of implementation and governance materials from Gavi and CEPI. Most of the 109 eligible documents were hosted on Gavi's website or published on the website of one of the other four lead organisations of COVAX in online repositories. Table 2 outlines the diversity of interviewees, including academics, civil society, and private sector representatives across multiple regions and presents characteristics of 23 interviewees, used to confirm or refute document review findings and seek explanations for why issues were addressed or not.

**TABLE 1:** Document characteristics.

Category	Type of document
Official documents	– Policies or policy directives – Strategies – Official statements and declarations – Position papers – Surveys
Implementation documents	– Organisational reports or evaluations – Financial analyses – Operational plans – Funding requests
Legal documents	– Laws – Regulations – Memorandums of understanding – Cooperation agreements
Working documents	– Meeting report or minutes – Committee reports – PowerPoint presentations – Mission reports
Media and communications	– Websites – News releases

**TABLE 2:** Summary of key informant interview characteristics.

Role	Code	Organisation	Location
Academic	AC‐1	Queen Mary University London	UK
	AC‐2	Georgetown University	USA
	AC‐3	University College London	UK
	AC‐4	University of Oslo	Norway
	AC‐5	University of Oslo	Norway
	AC‐6	Duke University	UK
	AC‐7	London School of Economics and Political Science	UK
	AC‐8	KEMRI Wellcome Trust	Kenya
	AC‐9	KEMRI Wellcome Trust	Kenya
	AC‐10	KEMRI Wellcome Trust	Kenya
Civil society	CV‐1	Médecins Sans Frontiers	UK
	CV‐2	Oxfam	UK
	CV‐3	Chatham House	UK
	CV‐4	Médecins Sans Frontiers	USA
Global health partnership	GP‐1	Gavi	USA
	GP‐2	WHO	Switzerland
	GP‐3	COVAX	Switzerland
	GP‐4	Global Fund	France
Private sector or vaccine manufacturer	PM‐1	Deloitte	UK
	PM‐2	Deloitte	USA
	PM‐3	Merck	France
	PM‐4	Deloitte	UK
	PM‐5	Merck	USA

### 3.1 Analytical Themes

Informed by Rushton and Williams' (2012) categorisation of global health policy, power configurations, competing policy frames, and underlying neoliberal paradigm, we generated two themes: (1) COVAX as a global vaccine equity policy process; and (2) competing technical and political solutions framing.

### 3.2 COVAX as a Global Vaccine Equity Policy Process

We generated four subthemes related to COVAX as an embodied process of global policy on vaccine equity: (i) GHP knowledge and leadership in COVAX creation; (ii) COVAX's income tiering; (iii) limited COVAX engagement with CSO and LMIC stakeholders; and (iv) lack of transparency and accountability in decision‐making in COVAX governance.

#### 3.2.1 GHPs' Knowledge and Leadership in COVAX Creation

While WHO provided normative global health leadership, two GHPs (CEPI and Gavi) were charged with leading the design of COVAX. This was confirmed by the documents, which showed how the process proceeded between May and August 2020. The three organisations were able to build on their collaboration in vaccine development, procurement, and delivery, each focussing on one of three workstreams: CEPI on development and manufacturing, Gavi on procurement and delivery, and WHO on policy and allocation [30, 31]. Interviewees saw Gavi and CEPI as a natural fit, given their experience in procurement and preparedness, and the lack of other capable organisations (AC1‐3, CV1‐4, PM1‐5).

CEPI was created to do this. This was disease x […]. CEPI and Gavi, they are both into vaccines, we need diagnostics and treatment […]. You can see where is the money, who can move money around. And then for instance, donors were ready to give loads of money, and they wanted to give it to initiatives they were confident were doing a good job, [and who were] more operational than WHO […] [Gavi and CEPI] have in their mandates to develop products, clinical trials, and procurement, and the things that WHO is not equipped with…

(CV‐1)

Interviewees generally agreed that increasing the WHO's public sector role before the pandemic would not have gained consensus. This was reflected in the long and only partly successful pandemic agreement negotiations. They noted a historical shift from the WHO's legally binding health equity initiatives to non‐binding GHP initiatives. Several (e.g., AC2‐5, CV1‐3, PM1‐3) noted that the WHO's limited capacity in vaccine R&D and severe underfunding hindered its role in policy, allocation, and norm‐setting during the pandemic response.

#### 3.2.2 COVAX's Income Tiering

COVAX initially proposed a needs‐based allocation model, termed the “Fair Allocations of Innovations for Pandemic Relief (FAIR) System” [30] but by September 2020, it had adopted income tiering to encourage HIC participation [32]. While some interviewees viewed this as a necessary political compromise, others felt it diluted equity goals [33].

Some suggested it reflected a concession to HICs and aligned with global health norms rather than challenging them.

And so, from the first discussions I had with […], he wanted to know if [we were] interested in getting on board with a project like that, which we were, but by the time the next iteration ‐ actually first paper ‐ came about, it was no longer that ambitious project and had already been diluted as something that would differentiate between rich and poor countries and the whole complicated set of that [which] COVAX started to crystallise. And I think from the beginning, the ambitions were compromised because the key people and institutions that were at the driving wheel were not ready to go for the big ambition.

(CV‐3)

Other interviewees (AC1, 5, GP2‐3, PM 3–5) argued that concessions were politically necessary because a unified platform accommodating all economies was not feasible and HICs would not have accepted the original terms. While there was agreement that COVAX's aim to achieve global vaccine equity was commendable, consensus suggested that focus should have been on LMICs rather than catering to HICs under the guise of solidarity.

#### 3.2.3 Limited COVAX Engagement With CSO and LMIC Stakeholders

On 4 June 2020, Gavi launched the COVAX AMC, consulting stakeholders including BMGF, CEPI, WHO, the World Bank, UNICEF, PAHO, McKinsey & Company, and various academics [34]. AMC countries and CSOs were not included in early consultations and had little influence on COVAX's structure. By July 2020, Gavi had defined the scope of countries included in the COVAX AMC but minimally involved their governments in decisions. In contrast, HICs significantly shaped COVAX's financial mechanisms. Some interviewees (AC1, 10 CV1‐4) suggested that CSOs were initially excluded because their demands were unlikely to be met.

After that initial convo with the then president of MSF, we said we were very interested, send us a concept note as soon as possible, happy to contribute etc. We never heard back, and I insisted several times […]. A few weeks later we received a concept note that was already very different. I think we were not invited to be part of the table because they knew what we would be standing for and fighting for.

(CV‐2)

LMIC and civil society actors began advocating for involvement in May 2020, before the AMC launch, but were not included in COVAX's governance structure until October 2020:

Even if African governments were involved, there is being involved and actually having a sway in how things are done, the latter was a problem. African countries were passive recipients of what became available, which makes it impossible to plan on how to roll out vaccines, whether or not they were involved, isn’t clear. What’s obvious is that as vaccines were being rolled out, African countries did not have a say

(AC‐10)

The lack of representation from LMICs in CEPI's governance and operations was highlighted as a significant issue, especially by those of our interviewees from LMICs (Table 2), whose perspectives underscore this governance gap. This aligns with findings from LMIC‐led analyses, such as that by Cranston, which detail how LMICs articulated their expectations and frustrations during pandemic treaty negotiations [35]. CEPI's Equitable Access Committee emphasised the need for broader engagement to build confidence, particularly with African and Asian scientists and populations [36]. Moreover, CEPI's Mid‐Term Review revealed it lacked representation from at‐risk countries, recommending that more inclusive expertise from industry and implementers in these countries could enhance governance and decision‐making [37]. This exclusion led to the creation of the African Vaccine Acquisition Trust (AVAT), which is supported by the African Union, to address imbalances in stakeholder representation. While it is too early to assess AVAT's impact on decision‐making and interactions with GHPs, there was consensus that the over‐representation of HIC interests needed rebalancing.

#### 3.2.4 Lack of Decision‐Making Transparency and Accountability in COVAX Governance

Most interviewees viewed COVAX's governance, modelled on Gavi's, as lacking transparency, inclusivity, and accountability. Meeting minutes revealed persistent confusion about the relationship between COVAX and the Gavi Board, underscoring the need for clearer governance structures [38]. Despite calls for reform, there was little evidence of willingness among COVAX leaders to adapt: ‘There is no desire for changes to be made to the COVAX governance model” [39]. Challenges such as manufacturing delays and geopolitics impacted vaccine delivery, further exacerbating existing tensions with LMIC stakeholders (AC 8–10, GP‐3, PM‐5).

COVAX was well intentioned but failed in part because of the novelty that arose from the pandemic, nobody could tell where we would be. By the time vaccines were arriving in African countries, most of the population had been exposed to natural infection, then we had an issue with planning around available vaccines—governments could not predict supply. This created questions around the utility in getting vaccinated with no demand.

(AC‐9)

While some interviewees emphasised the importance of public oversight and stakeholder engagement, others, particularly from the private sector, argued that contractual mechanisms already ensured accountability, framing transparency more as a matter of inclusivity than compliance.

Contracts are a red herring. If I am a pharmaceutical company and I have a contract with government x to deliver a certain amount of a vaccine by time y, that contract will have specifications that I am obligated to fulfil, or I don’t get paid. On the other side, the government or recipient will have a contracting officer, a procurement officer whose job it is to make sure that the government gets exactly what it bought. So, when people say there is no oversight and pharma companies aren’t accountable, I think this is nonsense, this is insulting to the governments and the other parties in those contracts […] The transparency question is more an issue of inclusivity and public participation.

(PM‐2)

### 3.3 Competing Technical and Political Solutions Framing

Our subthemes in relation to framing are the two competing global vaccine equity framings employed by policy actors: (i) achieving vaccine equity necessitates technical and financial mechanisms; and (ii) achieving global vaccine equity necessitates political solutions.

#### 3.3.1 Achieving Global Vaccine Equity Requires Technical Solutions and Financing Mechanisms

Documents revealed that Gavi and CEPI pursued vaccine equity primarily through technical and financial mechanisms, operating within existing intellectual property (IP) frameworks. Table 1 summarises the types of documents reviewed, including governance meeting minutes and board reports, which provided insight into these structural ambiguities. Their agreements included equity clauses but avoided discussions of IP waivers. While some stakeholders, particularly from industry and global health partnerships (PM1‐5; GP1‐3; AC3, 7), viewed patents as essential for innovation and private sector engagement, others, including civil society and academic voices, questioned this approach.

This is a sensitive issue in the industry, there are several bodies or individuals calling for IP waiver, but I think this is a simplistic view, not taking into consideration the huge amounts of other considerations. You need to protect the ability for organisations to invest in their innovation […] The [pharmaceutical] industry over time has always been used as a football, given the cost of healthcare and the cost of pharmaceuticals and some bad examples of profiteering which are well published in the sector. But the industry are on the side of angels with regard to what they are trying to do to promote health in the population. And again, I think it gets politicised and weaponised with the cost of bringing a drug to market.

(PM‐1)

They argued that the emphasis on corporate risk mitigation overlooked the substantial public investment in vaccine development and failed to address systemic barriers to equitable access. Interviewees highlighted that past successes in improving access, such as during the AIDS crisis, were driven by LMIC advocacy and generic production, not subsidies to large pharmaceutical firms.

Several interviewees (AC1, 5, CV 1–3) concurred that international pandemic responses should not solely ‘err on the side of pumping [manufacturers] with money [to] see what happens’ (AC‐5), noting that pandemics did not fit typical market failure scenarios when using tools like AMCs to help drive innovation. They criticised Gavi and CEPI's focus on corporate financial risks, which overlooked the significant public funding behind vaccine production and the pharmaceutical industry's capacity to absorb financial risks through profits. As one interviewee criticised the AMC model's effectiveness:

Gavi’s corporate subsidy‐based approach to global health, which is what it is, they just don’t use the word subsidy, but it’s exactly what they do and have been doing for 20 years, also always promises a diversification of corporations in the vaccine market, and that diversification has not happened for 20 years. If you look at their other AMCs they are giving scarce aid money to extremely profitable companies, without having any sense whatsoever whether that is enough money to spur innovation on their end, or without any evidence that this will diversify the market […] This does get us back to the historical study of how prices for generics in the AIDS crisis were brought down—it wasn’t through subsidies—it was done through generics and LMICs advocating for policy changes.

(AC‐5)

#### 3.3.2 Achieving Global Vaccine Equity Requires Political Solutions

Interviewees contended that increasing global manufacturing capacity was crucial for effective pandemic response, but views on implementation differed [40]. Some argued that Gavi and CEPI's focus on regional manufacturing overlooked broader health system issues and intellectual property concerns. Others questioned the expansion of the more technically complex mRNA vaccine manufacturing rather than other technologies.

Some interviewees highlighted how tensions over IP rules exacerbated manufacturing bottlenecks, with COVAX recognising too late the importance of regional manufacturing in the global pandemic response. This was evident when countries such as India prioritised vaccinating their populations or imposed export bans. Some interviewees noted that merely expanding global vaccine manufacturing would not tackle IP issues, which control vaccine availability through company‐held clinical data and marketing authorisations.

What I fear, that there is so much focus on manufacturing that we are no longer talking about IP. We’re not talking about how CEPI and Gavi are talking about setting up a network of producers which can be kept lukewarm so that they do not lose capacity, so that they can be mobilised in epidemic times […]. We often forget that you need to have the marketing authorisation […] With the support of the US and European governments, Moderna and Pfizer are now building factories in Africa. That is not going to change who controls where the vaccines are made available first, and at what price. Unless we take away the control from a few monopolies, we will be in exactly the same situation.

(CV‐1)

Another researcher from Kenya noted:

There has been a feeling of resentment in LMICs and that resentment was a bit more amplified, for example in Kenya, where in late 2020, that was when the trials for what would become the Oxford‐AstraZeneca vaccines were happening in Kilifi. Government agencies were pretty miffed at the idea that trials would be run in Kenya, and then vaccines would subsequently be unavailable.

(AC‐10)

Gavi and CEPI's board minutes acknowledge the need to expand manufacturing. However, interviewees differed on whether this increases vaccine access, with many (AC5‐9, CV1‐3, PM1, 3, 5) noting that increasing capacity does not resolve systemic bottlenecks hindering vaccine uptake in many LMICs. An interviewee highlighted these challenges:

Local manufacturing is a red herring, the conditions that are making it difficult to distribute vaccines in those countries are not going to change simply if you have a manufacturing plant which makes vaccines more available. The bottleneck is not supply, the bottleneck is now demand and infrastructure and distribution capacity and all the other things that go into a successful immunisation programme, and people are not paying enough attention to that […] The best example of this is Aspen in South Africa, they went on spec and built their own COVID‐19 manufacturing plant so they could provide generic vaccines and meet this demand. They built it a year ago and they haven’t had one order since.

(PM‐5)

Another noted:

Countries can pull together resources, for example, South Africa has vaccine manufacturing capacity, in Senegal there is the Institut Pasteur and they do vaccine research. It does not make sense [for every country to] set up their own vaccine production unit […], countries should harness their strengths and do things collectively, rather than individually.

(AC‐9)

Several argued that GHPs should instead concentrate on building health systems' capacity.

What are the parts of the global surveillance ecosystem that are really important and that need global coordination? How can organisations like Gavi and CEPI help to become part of those value chains…

(PM‐1)

A few interviewees (AC8, 9, CV1, GP4) highlighted the role of governments in favouring certain vaccine technologies, which they described as a political barrier to vaccine equity. They discussed the politicisation of ‘using the science’ within GHP operations, exemplified by stalled WHO approvals for the Russian Sputnik V vaccine following Russia's re‐invasion of Ukraine and resulting sanctions.

What the research world could do, is to begin to embrace different partnerships, If approached by [Contract Research Organisations] from different countries, as long as the phase 1 and phase 2 science is sound and published, it might make sense to give that a chance. For example, the current treatment of malaria is based off of very old Chinese medicine. What we are using now, the qinghaosu derivatives that were used for 1000s of years, is pretty efficacious and is the first line of management for malaria. In the same way, if the research world is blinded to other options, other than what is being pushed, we might lose the opportunity to find really efficacious molecules, which could work well in different populations. [To achieve this] there will be politics, from our [governments] or from funders, but these things can be navigated through diplomacy.

(AC‐10)

Traditionally, GHPs relied on Western pharmaceuticals, a policy that COVAX continued. However, interviewees questioned the dominance of Western companies in COVAX's response, noting that other nations with capacity, especially China, offered effective vaccine technologies but were marginalised.

The international system and partnerships have been relying on mRNA vaccines. If we look at the world, a huge proportion of the world geographically has been vaccinated with the Chinese vaccine, some parts of the world have been vaccinated with Sputnik, and we are currently learning that contrary to what has been pushed in our minds in the West, these are good vaccines […] What we are learning now is that three doses of Sinovac is equivalent to 3 doses of mRNA, this is now in the literature. I am saying this because from the beginning and because of the poor state, if not catastrophic state, of multilateralism these days, the dialogue was not global around global access and global R&D and the world was split from the beginning between the West—with the US industry leading, China, and to some extent Russia and India—4 blocks.

(GP‐4)

This is supported by Thambisetty et al., who analyse LMIC‐led efforts to challenge IP regimes, including the TRIPS waiver proposal by India and South Africa [41].

## 4 Discussion

### 4.1 Key Findings

Our findings confirm that COVAX operated similarly to previous GHPs [25]. As illustrated in Figure 1, the interplay of power dynamics and neoliberal paradigms influenced COVAX's design, governance and operational decisions. COVAX was initially a ‘buyer's club’ whose operations were underpinned by a commitment to global solidarity, an end‐to‐end approach to vaccine R&D, and leveraging both push and pull mechanisms [19]. Like earlier GHPs, COVAX did not challenge the political context or power dynamics shaped by Western HICs and their pharmaceutical industries. Therefore, it soon became another aid project based on the principle of charity for low‐income countries [17, 42]. COVAX shared vaccines, but not decision‐making power or production knowledge. To clarify why this shift occurred, we highlight competing power dynamics against a backdrop of assumptions and values that continue to underpin the vaccine innovation system.

### 4.2 Competing Global Vaccine Equity Framings

Rushton and Williams describe policy framing as an expression of agency, highlighting power relations ‐ with the likely success of competing policy frames influenced by power distribution [26]. We identified two main framings of global vaccine equity, that is one promoting technical and innovative financing solutions as favoured by GHPs and the private sector, and another promoting political solutions as favoured by (many) academics and CSOs.

GHPs' preferred framing and endorsement of a subsidy‐based approach aligned with their acceptance of donor‐driven expectations for measurable outcomes and the existing IP regime, reflecting a market‐friendly attitude or pragmatic adaptation to power dynamics in global health [19]. A study of how COVAX operated concluded that by conflating financial and public health risks, it privileged the former, ‘perpetuating the downsides of financialisation (e.g., heightened inequality, secrecy, governance complexity, ineffective and slow aid), whilst insufficiently realising its potential benefits (e.g., pandemic risk reduction, increased public access to emergency funding, indirect price control over essential goods and services)’ [19].

The second framing stressed the need for transparent discussions on manufacturing, IP ownership, inclusive decision‐making to address power imbalances, emphasising the unequal power in policy decision‐making processes, and the limited influence of those without material power [14, 43]. Academics and CSOs, despite lacking material power and being initially excluded from COVAX, wield ‘soft power’ through advocacy and persuasion, highlighting ongoing contestations in ensuring equitable participation in global health governance [44]. This is consistent with findings from our scoping review that Product Development Partnerships achieve an overall lack of stakeholder engagement [25].

### 4.3 Discursive, Resource, and Material Power

The technical and subsidy‐based approach to global vaccine equity is driven by the power dynamics and resources controlled by GHPs and the private sector, with significant influence from donor governments and pharmaceutical companies [45]. Reliance on subsidies raises questions about the effectiveness of this approach and the transparency of contract enforcement, despite some advocating for greater stakeholder involvement in decision‐making. Gavi and CEPI positioned themselves as technical leaders, leveraging donor trust and experience in vaccine delivery. However, their focus on technical solutions overlooked broader political challenges [46]. GHPs have become key players in global health governance, but their influence is seen as aligned with the interests of powerful donors and less with broadening the global discourse on equitable health solutions [47, 48]. The diminished role of WHO and its focus on normative functions, such as the Fair Allocation Framework, reflects these dynamics, in which financial and material power shape the priorities and approaches of global health policies [48].

### 4.4 The ‘Deep Core’ of Neoliberalism and Its Influence Over the Vaccine Equity ‘Policy Space’

Rushton and Williams contend that the ‘deep core’ of neoliberalism is a set of underlying assumptions and values shaping policies and institutions across domains, from global governance to individual self‐regulation, influencing economic, environmental and social spheres universally [26]. Neoliberalism in global health governance reflects decades of prioritising market‐based approaches, leading to commodification, privatisation and individual responsibility for health. Thus, authority has shifted from states and multilateral bodies to private, public‐private partnerships, and major foundations, reshaping how health policy and governance are structured and implemented worldwide [26, 48].

COVAX's design and implementation reflected what is termed the ‘deep core’ neoliberal principles in three key areas: governance, market preferences, and knowledge production [26]. Governance shifted away from multilateral oversight towards decentralised, donor‐driven structures, limiting LMIC and civil society participation. Market‐friendly policies prioritised private sector incentives and intellectual property protections, reinforcing existing power imbalances. Knowledge production was shaped by technocratic approaches that favoured measurable outputs over inclusive decision‐making and that treated vaccines as private property rather than (global) public goods. These dynamics were reflected in our findings, for example, the exclusion of LMIC voices from early governance discussions and the reliance on Western pharmaceutical firms despite viable alternatives, and the failure to challenge the prevailing IP rules. Together, these features entrenched a system that prioritised efficiency and innovation over equity and inclusion.

### 4.5 Implications and Lessons for Future Pandemic Mechanisms

These findings have implications for policy in four broad areas. The first relates to governance: strengthening transparency and inclusivity. Effective pandemic response mechanisms must be underpinned by governance structures that are transparent, inclusive, and representative of diverse global stakeholders, particularly those from LMICs and CSOs.

Equitable pandemic response depends on governance that is both transparent and inclusive. In the case of COVAX, many LMIC and civil society stakeholders were excluded from early decision‐making, a concern echoed by interviewees who felt their perspectives were marginalised. This lack of representation weakened the legitimacy of the initiative and limited its responsiveness to diverse needs. Future mechanisms must embed LMIC and CSO participation from the outset, ensuring their voices shape both strategic and operational decisions. They must, however, be tailored to different contexts, recognise diverse economic needs and prepare for rapid response capabilities in non‐pandemic times [22, 49, 50]. This requires collaboration with WHO regional offices and countries to understand individual health systems and tailor strategies accordingly [12], with Tupps et al. emphasising the importance of responses that draw on LMIC experiences [51].

Confusion over institutional roles, particularly between COVAX and the Gavi Board, was evident in governance documents and interviews. This ambiguity undermined accountability and created inefficiencies. Clear governance frameworks that define responsibilities and reporting lines are essential to avoid such overlap.

Transparency was another major concern. While some private sector representatives argued that contractual obligations ensured accountability, others stressed the need for public oversight. Publishing board minutes, funding allocations, and procurement decisions would help build trust and enable meaningful scrutiny.

Finally, stakeholder engagement must be institutionalised. The delayed inclusion of CSOs in COVAX's governance was seen as a missed opportunity. As CEPI's own reviews suggest, regular engagement with diverse stakeholders enhances legitimacy and improves policy outcomes. Strengthening governance in these ways will lay the foundation for more equitable and effective pandemic responses.

The second set relates to regional manufacturing capacity. Diversifying and decentralising vaccine manufacturing is essential to mitigate supply bottlenecks and reduce dependence on a few global producers. The COVID‐19 pandemic exposed the fragility of global supply chains, with limited production capacity concentrated in high‐income countries' facilities [15, 21, 22, 52]. Interviewees consistently emphasised the need for regional manufacturing hubs, citing initiatives like the African Vaccine Manufacturing Accelerator as promising models. This is echoed by Gloinson et al., who synthesise LMIC stakeholder perspectives on barriers and enablers to vaccine production [53].

However, regional manufacturing must be collaborative to avoid duplication and inefficiencies. Interviewees stressed that countries should pool resources and expertise rather than pursue isolated national strategies. As one interviewee noted, some institutions like the Institut Pasteur in Senegal already have strong research capacity, which could be leveraged through coordinated regional efforts.

Importantly, manufacturing strategies must be aligned with demand‐side realities. The case of Aspen Pharmacare in South Africa, where a COVID‐19 vaccine plant was built but received no orders, illustrates the risks of supply‐side overinvestment without adequate infrastructure and distribution planning. Interviewees warned that without robust delivery systems, increased production alone would not translate into improved access.

To be effective, future pandemic mechanisms must integrate manufacturing with broader health system planning and stakeholder engagement. While expanding production is necessary, it is not sufficient. As the next section will discuss, addressing intellectual property regimes is equally critical to unlocking broader participation and ensuring that manufacturing capacity can be fully utilised.

The third set relates to reform of intellectual property frameworks. This is critical for enabling equitable vaccine access and fostering sustainable innovation. During the COVID‐19 pandemic, restrictive IP regimes limited the ability of LMICs to manufacture and distribute vaccines, despite having the technical capacity. Interviewees emphasised the need to shorten IP protection periods during global health emergencies to facilitate technology transfer and local production. Public investment in vaccine development should come with conditions that ensure public benefit, including pre‐negotiated licencing agreements and public ownership of clinical trial data. This would allow governments and regional manufacturers to act swiftly without being constrained by proprietary barriers. Support for LMIC‐led initiatives, such as the TRIPS waiver proposal by India and South Africa, was also seen as essential to challenging monopolistic control and promoting global solidarity. Equitable access cannot be achieved if a handful of companies retain exclusive rights over life‐saving technologies. Addressing these legal and structural barriers is a necessary complement to expanding manufacturing capacity. However, even with improved IP access, vaccine equity will remain elusive without strong and adaptable health systems capable of delivering vaccines to those who need them most.

The fourth, and final set relates to strengthening health systems. This is vital to ensure that vaccines reach populations effectively and equitably. While manufacturing and distribution are critical, they must be supported by a robust delivery infrastructure and local capacity. Interviewees highlighted persistent bottlenecks in LMICs, such as limited cold chain logistics and workforce shortages, which hindered vaccine uptake even when doses were available. To address this, future pandemic mechanisms should collaborate closely with WHO regional offices to tailor responses to local health system constraints, ensuring that strategies are context‐specific and responsive to real‐world conditions. Investment in infrastructure and workforce development is essential—not only to improve vaccine delivery but also to build long‐term resilience. As emphasised by Tupps et al., drawing on LMIC experiences can inform flexible, locally grounded approaches that enhance preparedness and responsiveness. Without such tailored support, even well‐funded global initiatives risk failing to reach those most in need.

Taken together, these considerations highlight why GHPs must maintain a comprehensive approach to vaccine innovation, procurement, and delivery, supporting early R&D and technology transfer, especially during pandemics [54, 55].

## 5 Limitations

Our study has certain limitations. First, the small GHP and regional interviewee sample, predominance of UK universities and one private company may limit perspectives. We continued interviewing until data saturation, with no new topics raised. Second, our study design precluded assessment of technical effectiveness and causality, but these were not our focus. Lastly, COVAX is no longer operational, so we cannot know whether the changes we propose would have helped, although we hope they will inform any similar organisation in the next crisis.

## 6 Conclusions

COVAX largely replicated approaches adopted by earlier GHPs, working within the prevailing economic system and adopting market‐friendly policies to spur vaccine innovation to achieve global vaccine equity. This approach does not challenge the existing neoliberal economic system, which is not conducive to achieving global vaccine equity. This, we contend, is an important reason why COVAX underperformed. This should not detract from its accomplishments, which included major contributions to increased vaccine coverage in many low‐ and middle‐income countries (LMICs). However, gaps remained. COVAX endorsed the assumption that market‐friendly policies could foster innovation and ultimately address donor concerns about cost‐effectiveness. However, this has proven ineffective. The neoliberal paradigm hindered effective responses to political externalities and failed to address vaccine nationalism, which benefited donor and high‐income country (HIC) stakeholders but did not enhance global vaccine equity.

## Author Contributions

C.N., M.M., and N.H. conceived the study. C.N. collected and analysed data and drafted the initial manuscript. S.R., M.M., and N.H. contributed to theorisation and interpretation. N.H. revised for critical content. All authors approved the version for submission.

## Conflicts of Interest

MM is a member of the Advisory Committee of the Friends of the Global Fund Europe, from which he receives travel expenses. NH is a member of the Gavi Independent Review Committee, though this did not affect her interpretations. Other authors declare no conflict of interest.

## Supporting information


Supporting Information S1

## Data Availability

All data relevant to the study are included in the article.
