# Supplementary material for: ‘I Think From the Beginning, the Ambitions Were Compromised’: A Case Study of COVAX as Vaccine Equity Policy Operationalisation
Source: Int J Health Plann Manage. 2025 Sep 30;41(1):59–70. doi: 10.1002/hpm.70028 (PMC12794133; doi:10.1002/hpm.70028)
Supplement: Supplementary file 1 — Supporting Information S1 [file HPM-41-59-s001.docx]

**Interview guide The politics of COVID-19 vaccine equity: A case study of the COVAX Facility Initiative**

**Introduction**

READ: Interview [add ID code], without saying your name, please confirm that you have been informed about this study, your questions have been answered, you understand that if you wish to avoid a question or stop at any point you may do so, and that you are participating willingly.

1. Could you briefly summarise your professional role or experience in relation to COVAX?

**Topic 1: COVAX and its governance structure**

1. In what ways has COVAX achieved what it was set up to do? PROBE: has it achieved its goals of vaccinating the world, why/why not?
2. What are the main barriers facing COVAX?
3. COVAX has been critiqued as being a charity project for low and middle-income countries, do you think this is true, why/why not?
4. Why do you think many high-income countries have not procured vaccines via COVAX?
5. Why do you think there was limited CSO/LMIC involvement in the initial COVAX design process?
6. Why do you think many vaccines approved for emergency use are not included in the COVAX portfolio? PROBE: Why might manufacturers have chosen not to make their vaccines available via COVAX, despite creation of the No-Fault Compensation programme?

**Topic 2: how ideals of vaccine accessibility interact with the vaccine R&D ecosystem**

1. How could the current IP system act as a barrier to global accessibility of vaccines? PROBE: If it is a barrier, what is the role of COVAX in this?
2. Both Gavi and CEPI were created to address market failure, which has not appeared to be a problem during the COVID-19 pandemic, so do you think their preferred approaches to vaccine R&D and delivery were appropriate during the pandemic? PROBE: Why/why not?
3. In what ways have vaccine manufacturers dominated the current innovation system? PROBE: why is it supply led instead of demand led? How does this affect the likelihood of achieving global vaccine equity?
4. How can COVAX adapt to a vaccine system that favours suppliers and their interests?
5. COVAX adopts the GAVI procurement model, despite both facing very different problems, so do you think the GAVI model is appropriate during a global pandemic when countries compete for resources?
6. What is the role of public/private partnerships in a pandemic? PROBE: Are they effective within the context of countries competing for vaccines and resources?
7. Has there been an appropriate focus on global manufacturing capacity, why/why not?

**Topic 3: Responding to global health politics**

1. If countries are inherently self-interested, as international relations theory describes, how can COVAX address this self-interest and promote more multilateral cooperation?
2. In what ways do you think domestic politics have influenced how countries responded globally to COVID-19 or any other crisis?
3. How can non-state actors influence countries’ willingness to work together?
4. How does vaccine nationalism or diplomacy [*EXPLAIN both terms to the participant*], undermine the goal of global vaccine equity? PROBE: How can COVAX respond to this?

**Wrap-up**

Thank you for your time. We have reached the end of my questions. Is there anything else you would like to add or ask about that you think we didn’t discuss sufficiently?
